# Dumping syndrome following nissen fundoplication in an adult patient diagnosed by continuous online 13C/12C monitoring of ^13^C-Octanoic acid breath test "a case report"

**DOI:** 10.1186/1471-230X-11-98

**Published:** 2011-09-19

**Authors:** Meir Mizrahi, Gideon Almogy, Tomer Adar, Joseph Lysy

**Affiliations:** 1 Gastroenterology Institute, Hadassah Ein Karem Medical Center, Jerusalem, Israel

## Abstract

**Background:**

Nissen Fundoplication is a common surgical procedure performed in treating gastroesophageal reflux disease (GERD). Complications include dysphagia, gastric hypersensitivity, abnormal gastric motility, gas bloat syndrome and GERD relapse. Dumping syndrome may occur when a large volume of gastric content is delivered to the duodenum or jejunum, resulting in both gastrointestinal and vasomotor symptoms. Occasionally, dumping syndrome may be a complication in patients that have undergone nissen Fundoplication, especially in adults. The BreathID^® ^continuous online ^13^C-Octanoicoctanoic acid breath test detects variations of less than 1/100,000 in the ^13^CO_2_/^12^CO_2 _ratio in exhaled air.

**Case presentation:**

We report a case of a 38 year old male who was admitted and diagnosed with dumping syndrome following nissen Fundoplication, who was diagnosed using the BreathID^® ^continuous online ^13^C-Octanoic acid breath test.

**Conclusions:**

Early performance of a gastric emptying rate breath test in symptomatic patients, following upper GI tract surgery may help in the prediction or diagnosis of nissen Fundoplication complications such as dumping syndrome.

## Background

Initially described by Nissen in l956, gastric fundoplication was designed as a surgical procedure for the treatment of gastroesophageal reflux disease (GERD) [l]. Over the years, several modifications have been introduced, and in the early 1990's, progress in minimally invasive surgery facilitated the development of laparoscopic fundoplication. The laparoscopic approach offers equal functional results compared to the open approach [2-4]. As in any other types of invasive procedures, gastric fundoplication is associated with complications that include: dysphagia, gastric hypersensitivity, abnormal gastric motility, gas bloat syndrome and relapse of GERD symptoms [5]. Dumping syndrome has been documented as a complication of open fundoplication [6] and also in pediatric laparoscopic fundoplication [7,8].

The BreathID^® ^continuous online ^13^C-Octanoic acid breath test is a laser-like based technology that creates an infrared emission precisely matching the absorption spectrum of CO_2 _and can detect variations of less than 1/100,000 in the ^13^CO_2_/^12^CO_2_ratio measurement [9]. The system is based on the measurement of CO_2 _by molecular correlation spectroscopy. This test offers several advantages: It is an office-based, non-invasive tool for the assessment of substrate metabolism, does not involve a blood test and provides immediate results at the point-of-care [9].

A literature search yielded only a handful of reports on dumping syndrome as a complication of laparoscopic fundoplication in adults, but we think this is the first report regarding the diagnosis of post operative damping syndrome with this advanced technique [10].

We report the case of a 38-year-old patient who developed early dumping syndrome following nissen fundoplication. The BreathID^® ^^13^C-Octanoic acid breath test aided in confirming the diagnosis and he was successfully treated with gastrectomy and Roux-en-Y gastro-jejunostomy.

## Case presentation

A 38 year-old male was presented with abdominal pain, severe post - prandial weakness and diarrhea. Significant medical history showed a 10-year history of GERD symptoms most probably due to over weight (BMI = 35.2), for which he underwent laparoscopic nissen fundoplication 4 years earlier. The procedure consisted of a hiatal hernia repair and a short, 360-degree wrap over an intra-esophageal dilator. According to the operative report, both vagal nerves were identified and preserved.

Following surgery, the patient suffered from severe dysphagia and vomiting, and a 30 Kg weight loss over seven months. A barium test revealed stenosis/narrowing at the level of the esophageal gastric junction with recurrence of the hiatal hernia. One year after the laparoscopic procedure, the patient underwent exploratory laparotomy, seromyotomy of the distal esophagus and a redo floppy nissen fundoplication. Since then, the patient has neither GERD symptoms nor dysphagia.

Six months after the redo floppy nissen fundoplication the patient developed severe abdominal pain, bloating, borborygmy, faintness, weakness, palpitations, weight loss (BMI = 28.6) and urgency to defecate, which appeared 10-60 minutes after meals. The abdominal pain was not related to the meal size or content. He suffered from explosive chronic diarrhea with 3-6 bowel movements per day. He also complained of severe flatulence. Following defecation, the abdominal pain and bloating significantly improved. Several hours following meals he frequently suffered from severe weakness, palpitations and diaphoresis. He was seen by several gastroenterologists who attributed his complaints to irritable bowl syndrome (IBS), anxiety and depression. Indeed the patient developed nervousness and restlessness, making the correct diagnosis elusive.

At this point, the patient was referred to our clinic. The only abnormal finding on physical examination was slightly hyperactive, normal-pitched bowel sounds. The patient underwent a barium test which showed no unusual findings.. Esophagogastroduodenoscopy revealed a small gastric erosion which was biopsied, showing chronic inflammation, and his colonoscopy was normal. An abdominal Computer Tomography (CT) scan revealed many loops of small bowel attached to the anterior abdominal wall, without evidence of small or large bowel obstruction, and a slightly enlarged spleen.

**Figure 1:**
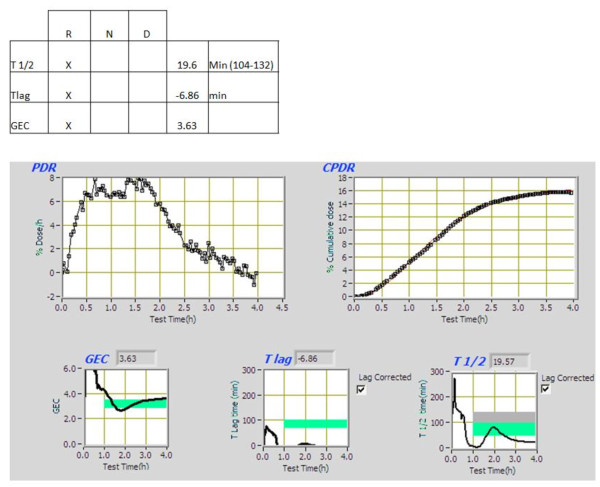
**Results of breath ID gastric emptying test**.

In view of the patient's complaints, we suspected that he was suffering from dumping syndrome. The patient underwent a ^13^C-Octanoic acid breath test (13C- Octanoic acid dose is 100 micro liters (92 mg), injected into the yolk of an egg. The egg yolk was then fired until congealed, and the white of the egg was added to the frying pan and the mixture was cooked until the entire mixture was congealed. The frying of the octanoic acid in the yolk provides a natural form of encapsulation of the 13-C octanoic acid in the solid component of the test meal. The egg was administered to a patient as sandwich inside two slices of white bread and with a glass of water for a total of approximately 250 kCal), which demonstrated rapid gastric emptying with T1/2 of 21.41 min (normal range 104-132 min) [Figure 1].

The patient was diagnosed to suffer from severe dumping syndrome which was unresponsive to dietary modifications. Medications such as Guar gum and acarbose were also not helpful. Octeotride treatment was declined due to need of chronic injections. It was therefore decided to offer the patient surgical treatment. Exploratory laparotomy revealed dense adhesions in the upper abdomen without additional gross pathology. The adhesions were divided, a subtotal gastrectomy was performed and gastro-intestinal continuity was reconstructed with a Roux-en-Y gastro-jejunostomy (10). His post-operative course was uneventful and he was discharged on the fifth post-operative day. After a one-year follow-up, he is pain-free and regained his weight, has well-formed bowel movements and is eating a regular diet.

## Discussion

Dumping syndrome is composed of 2 types, early (osmotic) and late (hypoglycemic). In both instances, a large volume of gastric content is delivered to the duodenum or jejunum resulting in symptoms of dumping. Where the anatomy of the pyloric sphincter has been altered by pyloroplasty, or bypassed by gastrojejunostomy, dumping syndrome can be explained by the direct unhindered passage of gastric content to the small bowel. Other mechanisms that do not include pyloric anatomy, include vagal damage [3,11] and disruption of gastric motility [5].

Symptoms can broadly be divided into gastrointestinal (nausea, cramping abdominal pain, and diarrhea) and vasomotor (sweating, tachycardia, palpitations, flushing, and dizziness). Early dumping usually appears 45 minutes after a meal, and seems to result from the passage of a large volume of osmotic material into the small bowel, causing an incursion of fluid from the intravascular space. The consequent reduction in circulating volume, combined with the release of vasoactive substances, such as vasoactive intestinal polypeptide (VIP), causes the symptoms.

In late dumping, similar symptoms can occur. However, they do not take place until 2 to 4 hours after a meal and the underlying mechanism is different. Rapid delivery of sugars/carbohydrates into the duodenum result in rising blood sugar levels which then cause an extreme serum insulin response with subsequent rebound hypoglycemia [9]. Symptoms of late dumping tend to be much more subtle and nonspecific than in the early syndrome, and the diagnosis may not always be considered. Confirmation is made by demonstrating a rebound hypoglycemia following a prolonged oral glucose tolerance test.

The use of surgical methods for treating dumping syndrome is uncommon, even after the medical treatment have failed. Our patient underwent a Roux en-y procedure known also as Lygidakis method published for the first time in 1994 [11].

The use of breath tests with ^13^C-Octanoic acid for the detection of rapid gastric emptying has been reported [12,13]. The use of BreathID^® ^continuous online ^13^C-Octanoic acid breath test system for the detection of the relationship between hypoglycemia and gastric emptying abnormalities in diabetic patients treated with subcutaneous insulin has been already published as well [14]. The fact that the test continuously displays the metabolized substrate, gives valuable data regarding the physiological performance of the stomach in real time. This could not be done if discreet breath samples were collected over time. Furthermore, the BreathID^® ^system can graphically display rapid gastric emptying and offers an advantage over the currently breath test methods being used, since it is an office-based, non-invasive does not involve a blood test and can provide an immediate result at the point-of-care.

## Conclusions

The experience accumulated in our institute confirmed that BreathID ^13 ^C octanoic acid breath test is reliable, easy to perform and can diagnose accurately gastric emptying abnormalities [15]. To the best of our knowledge, this is the first reported case of combined early and late dumping syndrome in an adult following nissen fundoplication. The ^13^C-Octanoic acid breath test can aid in the diagnosis of rapid gastric emptying and offers the unique advantage of being s an office-based, non-invasive tool for the assessment of substrate metabolism, providing an immediate result at the point-of-care. We therefore recommend early performance of gastric emptying rate breath tests in symptomatic patients, following upper GI tract surgery.

## Abbreviations

OBT: Octanoic acid breath test; GI: gastrointestinal; GERD: gastroesophageal reflux disease; VIP: vasoactive intestinal polypeptide.

## Consent

A consent form regarding this case report has been signed by the patient, and has been submitted separately.

## Competing interests

The authors declare that they have no competing interests

## Authors' contributions

MM: literature review, editing manuscript. AG: surgical operation, editing manuscript. TA: literature review, editing manuscript. LJ: final review, supervision of scientific content of manuscript. All authors have read and approved the final version of manuscript.

## Pre-publication history

The pre-publication history for this paper can be accessed here:

http://www.biomedcentral.com/1471-230X/11/98/prepub
