# Erratum: Dumping syndrome following nissen fundoplication in an adult patient diagnosed by continuous online 13C/12C monitoring of ^13^C-Octanoic acid breath test “a case report”

**DOI:** 10.1186/s12876-015-0280-8

**Published:** 2015-05-09

**Authors:** Meir Mizrahi, Gideon Almogy, Tomer Adar, Joseph Lysy

**Affiliations:** Gastroenterology Institute, Hebrew University-Hadassah Medical Center, POB12000, Jerusalem, IL 91120 Israel

## Erratum

We acknowledge that in the published work [1], text in the Background and Discussion sections was duplicated from S Kreckler, et al., 2006 Journal of the Society of Laparoendoscopic Surgeons [2] by mistake and we inadvertently failed to include this paper in the reference list. We would like to clarify that the case presented here is novel and has not been published before. We apologize for any inconvenience caused.
